# FlexSnap: Flexible Non-sequential Protein Structure Alignment

**DOI:** 10.1186/1748-7188-5-12

**Published:** 2010-01-04

**Authors:** Saeed Salem, Mohammed J Zaki, Chris Bystroff

**Affiliations:** 1 Department of Computer Science, North Dakota State University, Fargo, ND 58108, USA; 2 Department of Computer Science, Rensselaer Polytechnic Institute, 110 8th St, Troy, NY 12180, USA; 3 Department of Biology, Rensselaer Polytechnic Institute, 110 8th St, Troy, NY 12180, USA

## Abstract

**Background:**

Proteins have evolved subject to energetic selection pressure for stability and flexibility. Structural similarity between proteins that have gone through conformational changes can be captured effectively if flexibility is considered. Topologically unrelated proteins that preserve secondary structure packing interactions can be detected if both flexibility and Sequential permutations are considered. We propose the FlexSnap algorithm for flexible non-topological protein structural alignment.

**Results:**

The effectiveness of FlexSnap is demonstrated by measuring the agreement of its alignments with manually curated non-sequential structural alignments. FlexSnap showed competitive results against state-of-the-art algorithms, like DALI, SARF2, MultiProt, FlexProt, and FATCAT. Moreover on the DynDom dataset, FlexSnap reported longer alignments with smaller *rmsd*.

**Conclusions:**

We have introduced FlexSnap, a greedy chaining algorithm that reports both sequential and non-sequential alignments and allows twists (hinges). We assessed the quality of the FlexSnap alignments by measuring its agreements with manually curated non-sequential alignments. On the FlexProt dataset, FlexSnap was competitive to state-of-the-art flexible alignment methods. Moreover, we demonstrated the benefits of introducing hinges by showing significant improvements in the alignments reported by FlexSnap for the structure pairs for which rigid alignment methods reported alignments with either low coverage or large *rmsd*.

**Availability:**

An implementation of the FlexSnap algorithm will be made available online at http://www.cs.rpi.edu/~zaki/software/flexsnap.

## Background

The wide spectrum of functions performed by proteins are enabled by their intrinsic flexibility [1]. It is known that proteins go through conformational changes to perform their functions. Homologous proteins have evolved to adopt conformational changes in their structure. Therefore, similarity between two proteins which have similar structures with one of them having undergone a conformational change will not be captured unless flexibility is considered.

The problem of flexible protein structural alignment has not received much attention. Even though there are a plethora of methods for protein structure comparison [2-8], the majority of the existing methods report only sequential alignments and thus cannot capture non-sequential alignments. Non-sequential similarity can occur naturally due to circular permutations [9] or convergent evolution [10]. The case is even harder for flexible alignment since only two methods, FlexProt [11], and FATCAT [12] report flexible alignments. Nevertheless, both methods are inherently limited to *sequential *flexible structural alignment because both methods employ sequential chaining techniques. The complexity of protein structural alignment depends on how the similarity is assessed. Kolodny and Linial [13] showed that the problem is NP-hard if the similarity score is distance matrix based. Therefore, over the years, a number of heuristic approaches have been proposed, which can mainly be classified into two main categories: dynamic programming and clustering.

Dynamic Programming (DP) is a general paradigm to solve problems that exhibit the optimal substructure property [14]. DP-based methods, Structal [15] and SSAP [16], construct a scoring matrix *S*, where each entry, *S*_*ij*_, corresponds to the score of matching the *i*-th residue in protein A and the *j*-th residue in protein *B*. Given a scoring scheme between residues in the two proteins, dynamic programming finds the global alignment that maximizes the score. DP-based methods suffer from two main limitations: first, the alignment is sequential and thus non-topological similarity cannot be detected, and second, it is difficult to design a scoring function that is globally optimal [13]. In fact, structure alignment does not have the optimal substructure property, therefore DP-based methods can find only a suboptimal solution [17]. The other category of alignment methods, the Clustering-based methods, DALI [2], SARF2 [4], CE [5], SCALI [7], and FATCAT [12], seek to assemble the alignment out of smaller compatible (similar) element pairs such that the score of the alignment is as high as possible [18]. Two compatible element pairs are consistent (can be assembled together) if the substructures obtained by elements of the pairs are similar. The clustering problem is NP-hard [19], thus several heuristics have been proposed. The approaches differ in how the set of compatible element pairs is constructed and how the consistency is measured. Both SARF2 and SCALI produce non-sequential alignments.

The two main flexible alignment methods, FlexProt [11] and FATCAT [12], work by clustering (chaining) aligned fragment pairs (AFPs) and allowing flexibility while chaining, by introducing hinges (twists). FlexProt searches for the longest set of AFPs that allow different number of hinges. It then reports different alignments with different number of hinges. The FATCAT method works by chaining AFPs using dynamic programming. The score of an alignment ending with a given AFP is computed as the maximum score of connecting the AFP with any of alignments that end before the AFP. A penalty is applied to the score to compensate for gaps, root mean squared deviation (*rmsd*), and hinges. A third method, which can handle flexible alignments, is the HingeProt [20] method. HingeProt first partitions one of the two proteins into rigid parts using a Gaussian-Network-Model-based (GNM) approach and then aligns each rigid region with the other protein using the MultiProt [6] method. HingeProt uses the MultiProt algorithm in the sequential mode and thus does not report flexible non-sequential alignments. Therefore, the accuracy of the HingeProt approach depends on the accuracy of identifying the rigid domains which is a hard problem as the best known method, HingeMaster [21], has a sensitivity of only 50%.

To address the limitations of exisiting algorithms we propose FlexSnap, a greedy algorithm for flexible sequential and non-sequential protein structural alignment (the name of the algorithm is a non-sequential permutation of the bold letters in **Flex**ible **n**on-**S**equential **p**rotein **a**lignment). The algorithm assembles the alignment from the set of AFPs and allows non-sequential alignments and hinges. We demonstrate the effectiveness of FlexSnap by evaluating its alignments' agreement with manually curated non-sequential alignments. Moreover, FlexSnap shows competitive results on the FlexProt dataset when compared to the main flexible alignment methods, FlexProt and FATCAT.

## Methods

**Figure 1:**
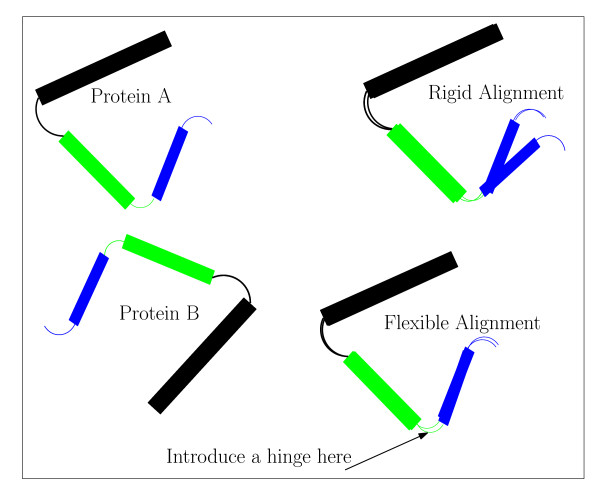
**Flexible Structural Alignment**. The Figure shows proteins *A *and *B *which have 3 similar structure fragments. A rigid alignment (top right) is not able to align the blue fragment, but a flexible alignment (bottom right) can do this easily by introducing a hinge between the rigid block (the black and green fragments) and the blue fragment. As we assemble the alignment from well-aligned pairs, we introduce hinges to get a longer alignment and smaller *rmsd*.

The main idea of the FlexSnap approach is to assemble the alignment from short well-aligned fragment pairs, which are called AFPs. As we assemble the alignment by adding AFPs, introducing hinges when necessary. Figure 1 shows how the alignment is constructed from smaller aligned fragment pairs. When chaining a fragment pair to the alignment, we choose the fragment that has the highest score when joined with the last rigid region in the alignment. The score rewards longer alignments with small *rmsd *and penalizes large *rmsd*, gaps, and the introduction of hinges. In the next subsections, we provide a detailed discussion of the FlexSnap algorithm.

### AFPs Extraction

Let *A *= {*A*_1_, *A*_2_,..., *A*_*n*_} and *B *= {*B*_1_, *B*_2_,..., *B*_*n*_} be two proteins with *n *and *m *residues respectively, where *A*_*i *_∈ ℜ^3 × 1 ^(similarly *B*_*i*_) represents the 3D coordinates of the *C_α _*atom of the *i*-th residue in protein *A*. The first step in FlexSnap is to generate a list of aligned fragment pairs (AFPs):

Each AFP, (*i*, *j*, *l*), is a fragment that starts at the *i*-th residue in *A *and *j*-th residue in *B *and it has a length of *l *residues. An AFP is formally represented as a set of *l *equivalenced pairs between the two proteins, and given as:

where (*A*_*i*_, *B*_*j*_) indicates that the *i*^*th *^residue of protein *A *is paired with the *j*^*th *^residue of protein *B*, and *l *is AFP's length. Each AFP must satisfy a user-defined similarity constraint. In FlexSnap, we employ the root mean square deviation as the similarity measure, i.e., *rmsd*(*i*, *j*, *l*) ≤ ε. Moreover, we require that the length of the AFP be at least *L*, i.e., 3 ≤ *L *≤ *l*. Furthermore, we define  and  to be the beginning and end of the AFP_*k *_along the backbone of protein *B*. For example, for a triplet AFP_*k *_= (*i*, *j*, *l*) and protein *A*,  = *i *and  = *i *+ *l *- 1.

The number of possible AFPs can be as large as *O*(*n*^3^). The set of all AFPs can be obtained by iterating over all the triplets (*i*, *j*, *l*),

and for each triplet checking if the *rmsd*(*i, j, l*) ≤ ε. The *rmsd *of a fragment of length *l *can be obtained in *O*(*l*) [22]. A naive implementation that iterates over all the triplets (*i, j, l*) to obtain the set of all the AFPs would have an *O*(*n*^4^) time complexity. However, by observing that the *rmsd *of the AFP (*i, j, l + 1*) can be computed incrementally from the *rmsd *of AFP (*i, j, l*) in constant time, the set of aligned fragment pairs (AFPs) can be obtained in *O*(*n*^3^) time complexity [11].

The main idea to incrementally compute the *rmsd *is to simplify the *rmsd *formula. Given two sets, *A *and *B*, of *N *points each, the root mean square deviation (*rmsd*) is calculated as [23]:

where *A' *and *B' *denote the points after recentering, i.e., , and the *d*_*i*_'s are the singular values of *C *= *A'B'*^*T*^, which is a 3 × 3 covariance matrix given as:

In rare cases when the determinant of *C *is negative, then *d*_3 _= -1 * *d*_3_. Equation (1) can be simplified as:

It is clear that all the terms used in equation (3) can be updated in constant time, and thus computing the *rmsd *for *N *+ 1 points requires constant time if we have all the terms evaluated for the first *N *points. Therefore computing the *rmsd *for AFP(*i, j, l*) for all values of *l*'s (for a given *i *and *j *) requires only *O*(*n*) time. Thus, the total time complexity for the seeds extraction step is *O*(*n*^3^) ...

### Flexible Chaining

The second step in FlexSnap is to construct the alignment by selecting a subset of the AFPs. Given a set of AFPs, *P*, obtained in the AFPs extraction step, we are interested in finding a subset of AFPs, *R *⊆ *P*, such that all the AFPs in *R *are mutually non-overlapping and the score of the selected AFPs in *R *is as large as possible. At one hand, we want to get as large an alignment as possible, while on the other hand, we want to minimize the number of hinges and gaps. Therefore, our goal is to optimize a score that rewards long alignments with small *rmsd*, and penalizes the introduction of hinges and gaps.

**Figure 2:**
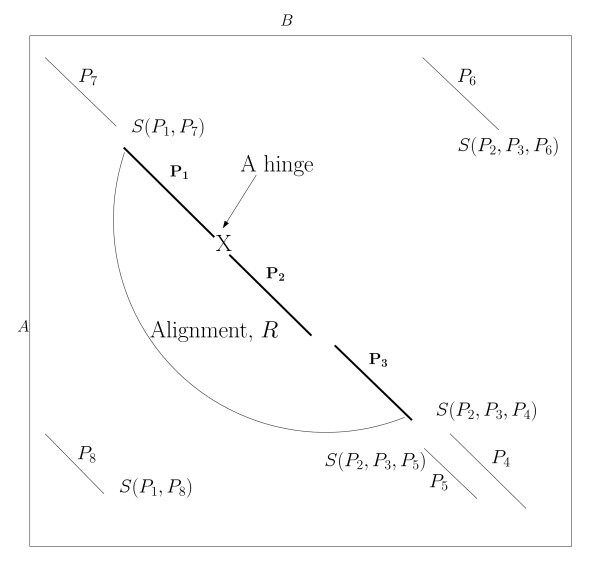
**Flexible Structural Alignment by AFPs chaining**. When extending the alignment *R *= {*P*_1_, *P*_2_, *P*_3_}, the score of extending *R *with each AFP is computed and we extend the alignment with the AFP that gives the best score. The score *S*(*P*_4_, *P*_2_, *P*_3_)) indicates the score of adding *P*_4 _to the region composed of *P*_2 _and *P*_3_.

The set of AFPs can be thought of as runs in an *n × m *matrix *S*, where *n *and *m *are the sizes of proteins *A *and *B*, respectively (see Figure 2). We define a precedence relation, ≺, between two AFPs such that *P*_*i *_≺ *P*_*j *_if *P*_*i *_appears either in the upper or lower left quadrant of *P*_*j*_, i.e.  and , or  and  (recall that  and  denote the beginning and end, respectively, of AFP *P*_*i *_in protein *A*). Generally speaking, we say that two AFPs, *P*_*i *_and *P*_*j*_, can be chained if they do not overlap, i.e., *P*_*i *_≺ *P*_*j *_or *P*_*j *_≺ *P*_*i *_As depicted in Figure 2, *P*_7 _and *P*_8 _can be chained to *P*_1_.

For sequential chaining, we define a sequential precedence relation, ≺_*s*_, such that *P*_*i *_precedes *P*_*j *_(written as *P*_*i *_≺_*s *_*P*_*j*_) if *P*_*i *_appears strictly in the upper left quadrant with respect to *P*_*j*_, i.e.  and . Two AFPs *P*_*i *_and *P*_*j *_can be sequentially chained together if *P*_*i *_≺_*s *_*P*_*j *_or *P*_*j *_≺_*s *_*P*_*i*_. In Figure 2, *P*_7 _and *P*_2 _can be sequentially chained to *P*_1_. An AFP, *P*_*i*_, can be chained to an alignment *R*, denoted as (*R *→ *P*_*i*_), if it does not overlap with any AFP in *R*. In Figure 2, *P*_7_, *P*_4_, and *P*_5 _can be sequentially chained to *R *which consists of AFPs {*P*_1_, *P*_2_, *P*_3_}; and both *P*_6 _and *P*_8 _can be non-sequentially chained to *R*. Next, we shall introduce our solution for the general flexible chaining problem.

### The FlexSnap Approach

The goal of chaining is to find the highest scoring subset of AFPs, i.e., *R *⊆ *P*, such that all the AFPs in *R *are mutually consistent and non-overlapping. The problem of finding the highest scoring subset of AFPs is essentially the same as finding the maximum weighted clique in a graph *G *= (*V, E, w*) where the set of vertices *V *represent the set of AFPs, each vertex *v*_*i *_has a weight equal to the score of the AFP, *w*(*v*_*i*_) = *S*(*P*_*i*_), where the score of an AFP *P*_*i*_, *S*(*P*_*i*_), could be its length or some other combination of length and *rmsd*. There is an edge (*v*_*i*_, *v*_*j*_) ∈ *E *if the AFPs *P*_*i *_and *P*_*j *_do not overlap and are consistent (can be joined with small *rmsd *or have similar rotation matrices).

The problem of finding the maximum weighted clique in a graph is computationally expensive; it is NP-hard [19]. Thus, we propose a greedy algorithm to find an approximate solution for the chaining problem. The main idea is to start building the alignment from an initial AFP and to add AFPs to the alignment. We start the alignment by selecting the longest AFP, then we iteratively add new AFPs to the alignment as long as the newly added AFP improves the score of the alignment. Given an alignment, *R*, we add to it the AFP that contributes most. We keep growing the alignment until no more AFPs can be added. The contribution of an AFP to the alignment is scored by how consistent the AFP is with the alignment and how good the AFP is. When adding an AFP to an alignment, we reward longer AFPs with smaller *rmsd*, and we penalize for gaps, inconsistency, and hinges. The penalty takes into consideration: 1) the number of gaps introduced; 2) the increase in *rmsd *when combining two or more AFPs; 3) the introduction of new hinges.

As depicted in Figure 2, the scores of extending the alignment, *R*, with *P*_4_, *P*_5_, *P*_6_, *P*_7_, or *P*_8 _are computed and the AFP with the best score is added to the alignment. When measuring the score of adding an AFP to the alignment, we actually measure the score of adding the AFP to the last rigid region, and not just to the last fragment, in the alignment. In Figure 2, the score of adding *P*_4 _to *R *is the score of adding *P*_4 _to the region composed of *P*_2 _and *P*_3_. Since *P*_2 _and *P*_3 _together form a rigid sub-alignment (as we can see there is no hinge between them). When adding *P*_7 _to *R*, the score of adding *P*_7 _to the region composed only of *P*_1 _is computed.

**Figure 3:**
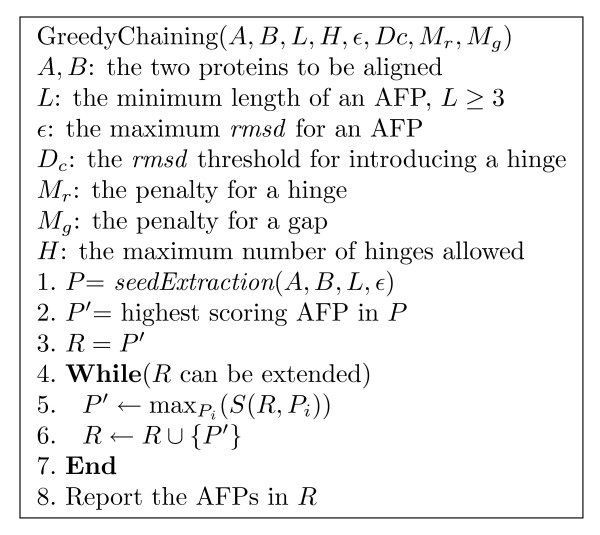
**A greedy AFP chaining algorithm**. A greedy algorithm for AFP chaining. The algorithm iteratively chooses an AFP to add to *R *(lines 4-7) until no more AFPs can be added, or the best score of adding an AFP to *R *is negative.

Figure 3 shows the pseudo-code for the greedy chaining algorithm used in FlexSnap. Since the chaining is a greedy algorithm, we run the algorithm *K *times starting from the *K *highest scoring non-overlapping AFPs and we report the alignment with the best score.

#### Alignment Extension Score

Next, we will discuss how we extend a partial alignment with the next best AFP. More specifically, given an alignment *R*, the next AFP to chain to the alignment is the one that maximizes the following scoring function:

where *R *→ *P*_*i *_indicates that *P*_*i *_does not overlap with *R*, and *S*(*R, P*_*i*_) is the score of chaining *P*_*i *_to *R*. The score, *S*(*R, P*_*i*_), is a combination of the weight of the AFP, *W*(*P*_*i*_), and the penalty of extending *R *with *P*_*i*_, *C*(*R *→ *P*_*i*_). The score is defined as follows:

where *C*(*R *→ *P*_*i*_) is the penalty incurred when connecting *P*_*i *_to *R*, and *W*(*P*_*i*_) is the score of the AFP itself. The scoring function rewards longer AFPs with small *rmsd *and penalize gaps and hinges. If the addition of an AFP *P*_*i *_to the alignment results in a large *rmsd*, then we introduce a hinge only if *W*(*P*_*i*_) is large enough to compensate for the penalty incurred. A similar approach for penalizing gaps and hinges was used in the FATCAT method [12]. Though their score and cost functions are different, and they do not consider rigid regions as we do in FlexSnap when connecting an AFP to the alignment. The score of connecting *P*_*i *_to *R *is defined as follows:

where *M*_*g *_is the penalty for a gap, *M*_*r *_is the maximum penalty for a hinge, and  is the *rmsd *of connecting *P*_*i *_to the last rigid region in *R*. If  increases above a user-defined threshold, *D*_*c*_, we introduce a hinge and the penalty is maximum; if not, the penalty is proportional to how far the *rmsd *value is from *ε *(maximum *rmsd *for an AFP). Moreover, we allow only a maximum number of *H *hinges. The score for an AFP is a function of its length and *rmsd*. The score is the length of the AFP, *L*(*P*_*i*_), plus a contribution of the *rmsd *of the AFP, *rmsd*(*P*_*i*_), to the score, and is given as:

The complexity of the chaining algorithm depends on the number of AFPs, M, that the two structures have. In the worst case, *M *could be close to *n*^3^, but in practice it is much less, i.e., *M *≤ *n*^2^. The complexity of the algorithm is *Mlog*(*M*) + k * M * *n*, where k is the number of AFPs in the final solution and *n *is the size of the larger protein.

#### Sequential Flexible Chaining

**Figure 4:**
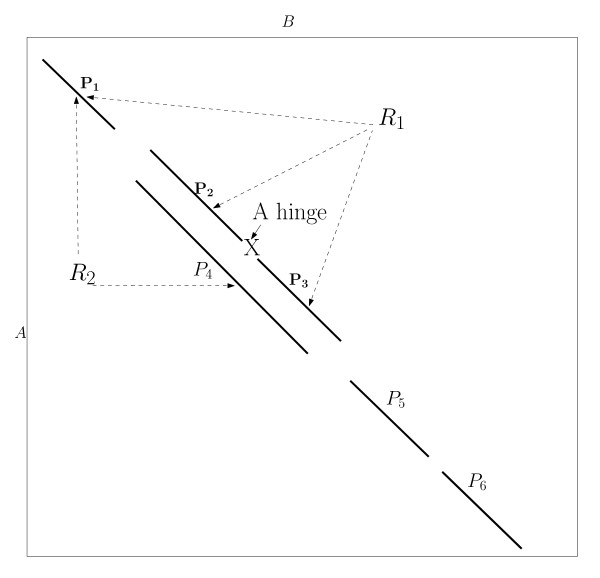
**A greedy sequential AFP chaining algorithm**. A greedy sequential algorithm for AFP chaining. When encountering the beginning of an AFP, the algorithm computes the scores of adding the AFP to the alignments in the upper left corner and the AFP is chained to the alignment with which it gives the highest score.

The above general chaining algorithm reports both sequential and non-sequential alignments. In the results section, we demonstrate that the quality of its non-sequential alignments is competitive to state-of-the-art non-sequential alignment methods. However, for sequential flexible alignment, there are more efficient chaining algorithms, namely the approach proposed by the FATCAT algorithm. The FATCAT algorithm follows a dynamic programming approach for chaining the AFPs. In FATCAT, the score of an alignment ending with an AFP, *P*_*i*_, is defined in terms of the score of *P*_*j*_'s and the connection cost of *P*_*i *_with these *P*_*j*_'s such that *P*_*j *_precedes *P*_*i *_(*P*_*j *_≺_*s *_*P*_*i*_). More specifically, FATCAT defines the score of the alignment that ends with *P*_*i *_as follows:

where *C*(*P*_*j *_→ *P*_*i*_) is the penalty incurred when connecting *P*_*i *_to the alignment that ends with *P*_*j *_and it is similar to the penalty function used in the general chaining and *W*(*P*_*i*_) is the score of the AFP itself. We propose an approach that is similar in spirit to FACTCAT, however, it is different in two important aspects. The first aspect is the optimality of the alignment reported by FATCAT. The main issue here is that the scoring function has an *rmsd *term since *W*(*P*_*i*_) is a function of the length of *P*_*i *_and its *rmsd*. Therefore, *S*(*P*_*i*_) cannot be optimal because we do not know of a scoring function that involves the *rmsd *value that is additive and optimal (*rmsd *score is not a metric since it does not satisfy the triangular inequality property). Therefore, the optimality of FATCAT alignments is not guaranteed since the sub-optimality property of the dynamic programming does not hold if the score incorporates an *rmsd *term. In Figure 4, let the optimal alignment be {*P*_1_, *P*_4_, *P*_5_, *P*_6_}, the sub-optimality property requires that {*P*_1_, *P*_4_, *P*_5_} is also optimal, and it is the best alignment that ends with *P*_5_. This is not necessarily true in structural alignment, because it could happen that the alignment {*P*_1_, *P*_2_, *P*_3_, *P*_5_} is better {*P*_1_, *P*_4_, *P*_5_}. In general, the flexible structural alignment does not exhibit the optimal substructure that would justify the use of dynamic programming.

In FlexSnap, we follow a similar approach as the approach presented in [24] for chaining substrings. In the original algorithm, once we reach the end of a substring (segment), *P*_*i*_, we delete all the solutions that end with *P*_*j*_'s whose ends are lower and to the left of the endpoint of *P*_*i *_and *S*(*P*_*j*_) <*S*(*P*_*i*_). For the segments shown in Figure 4, let *S*(*P*_3_) >*S*(*P*_4_), once we encounter the end of *P*_3_, we should delete the solution that ends with *P*_4_. When we encounter *P*_5_, we know that the best solution it can be chained to ends with *P*_3_. This approach works fine for regular chaining problems (like strings). However for the structural alignment problem, this approach does not yield the optimal alignment since the problem does not exhibit the optimal substructure property. Therefore, in FlexSnap, once we reach the end of an AFP, *P*_*i*_, we do not delete all solutions that end with *P*_*j*_'s which are to the left and below *P*_*i*_; instead we only delete the ones that have very low scores as compared to *S*(*P*_*i*_). Though not optimal, this approach gave better results for sequential chaining than the pure greedy approach presented in the previous section.

The second aspect where FlexSnap is different from FATCAT is that in FATCAT *C*(*P*_*j *_→ *P*_*i*_) is the connection cost of *P*_*i *_and *P*_*j *_while in FlexSnap it is the connection cost of *P*_*i *_to the rigid region that contains *P*_*j*_. In FATCAT, if *P*_*j *_belongs to a rigid region and the connection cost of *P*_*i *_with *P*_*j *_is small, *P*_*i *_will be added to the same rigid region as *P*_*j *_even though *P*_*i *_might not be consistent with other AFPs in the same region. In Figure 4, if we were connecting *P*_5 _to *R*_2 _that ends with *P*_4_, FATCAT would compute the connection cost *C*(*P*_5_, *P*_4_) but FlexSnap would compute *C*((*P*_1_, *P*_4_) → *P*_5_) since *P*_4 _belongs to the rigid region that contains *P*_1_. In FATCAT, when connecting *P*_5 _to *R*_2_, we might get the conclusion that there is no need to introduce a hinge and thus *P*_5 _belongs to the same rigid region as *P*_1 _and *P*_4_. This may lead to a large *rmsd *when we report the alignment since we did not check if *P*_5 _is consistent with *P*_1_. However, when FlexSnap adds *P*_5 _to the same rigid region as *P*_4 _and *P*_1_, it will not harm the final *rmsd *when we report the alignment as FlexSnap ensures that all the segments in the same rigid region are consistent. In the results section, we investigate how computing the connection cost with the whole rigid region as opposed to the last segment in the rigid region affects the quality of the alignment. For some structure pairs, considering the whole rigid region in computing the connection cost resulted in significant improvements.

## Results and Discussion

To assess the quality of FlexSnap alignment compared to other structural alignment methods, we evaluated the agreement of the methods' alignments with reference manually-curated alignments. We compared FlexSnap against sequential methods (DALI [2] and CE [5]), non-sequential methods (SARF2 [4], MultiProt [6], and SCALI [7]), and flexible sequential alignment methods (FlexProt [11] and FATCAT [12]). Finally, we analyzed the flexibility on the DynDom dataset [25], which is a comprehensive and non-redundant dataset of protein domain movements.

All the experiments were run on a 1.66 GHz Intel Core Duo machine with 1 GB of main memory running Ubuntu Linux. The chaining algorithm is efficient and its running time varies from 1 second to a minute depending on the size of the proteins. We used the corresponding web server for most of the other alignment methods. The optimal values for the different parameters were found empirically such that they give the best agreement with manually curated alignments; we used *L *= 8, *ε *= 2 Å, *D*_*c *_= 3 Å, *α *= 0.5, *M*_*r *_= -10, *M*_*g *_= -1, and *H *= 3 (see Figure 3).

### Non-Sequential Alignments

We used the reference alignments for the structure pairs which have circular permutation in the RIPC dataset [26]. The RIPC set contains 40 structurally related protein pairs which are challenging to align because they have indels, repetitions, circular permutations, and show conformational flexibility [26]. There are 10 pairs in the RIPC dataset that have circular permutation. Since the structure pairs have non-sequential alignments, to be fair, we only compare with algorithms that can handle non-sequentiality. However, we report the average agreement for some sequential methods as well. The agreement of a given alignment, *S*, with the reference alignment, *R*, is defined as the percentage of the residue pairs in the alignment which are identically aligned as in the reference alignment (*I*_*S*_) relative to the reference alignment's length (*L*_*R*_), i.e., *A*(*S*, *R*) = (*I*_*S*_/*L*_*R*_) × 100. Table 1 shows the agreements of four different methods with the reference alignments in the RIPC dataset. The results show that FlexSnap is competitive to state-of-the-art methods for non-sequential alignment. In fact, it has the highest average agreement (79%) among the methods shown. The average agreement of most of the sequential alignment methods we compared with were drastically lower: DALI [2] (40%), CE [4](36%), FATCAT [12](28%), and LGA [27](38%).

**Table 1:** Comparison of SARF, MultiProt, SCALI, and FlexSnap on the RIPC dataset.

SCOPID		SARF			MultiProt			SCALI			FlexSnap		
**Pro1**	**Pro2**	**size**	***rmsd***	**A**	**size**	***rmsd***	**A**	**size**	***rmsd***	**A**	**size**	***rmsd***	**A**

d1nkl__	d1qdma1	67	2.21	92	67	1.82	68	62	1.94	69	73	2.39	100
d1nls__	d2bqpa_	212	1.50	83	213	1.03	100	195	1.62	83	210	2.81	83
d1qasa2	d1rsy__	109	2.27	65	107	1.24	93	98	1.92	82	111	1.73	100
d1b5ta_	d1k87a2	171	2.63	63	144	2.04	0	159	3.38	0	177	2.99	50
d1jwyb_	d1puja	115	2.43	83	108	1.81	92	110	4.60	83	116	2.61	92
d1jwyb_	d1u0la2_	97	2.02	100	103	1.86	91	91	4.52	90	96	2.82	100
d1nw5a_	d2adma	129	2.52	85	130	2.11	92	132	3.73	84	128	2.91	100
d1gsa 1_	d2hgsa1	73	2.59	20	74	1.56	40	69	3.23	40	73	2.81	20
d1qq5a_	d3chy__	88	2.39	67	82	1.97	67	52	2.08	66	93	2.94	67
d1kiaa_	d1nw5a_	146	2.48	83	153	1.85	75	138	3.99	75	141	2.69	75

Avg.	Agreement			74			72			67			79

Three values are reported for each alignment: its length, its *rmsd*, and A which is its agreement with the reference alignment in the RIPC dataset

**Figure 5:**
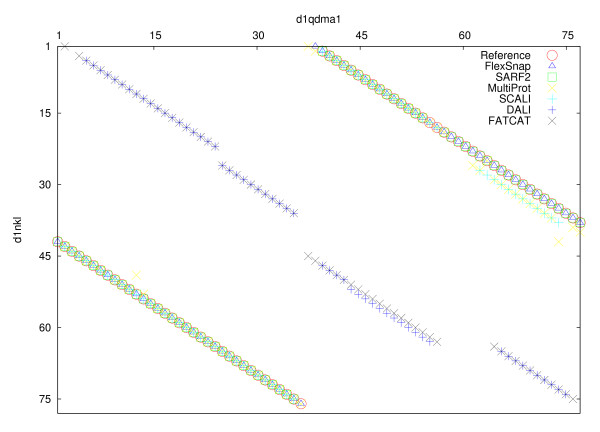
**Comparison of the agreements of the alignments with one structure pair from the RIPC dataset**. Comparison of the agreement between the reference alignment and 6 other alignment methods on the structure pair of prophytepsin(d1qdma1) and nk-lysin(d1nkl__). Residue positions of d1qdma1 and d1nkl__ are plotted on the x-axis and y-axis, respectively. Note: the reference alignment pairs are shown in circles. The SARF, MultiProt, SCALI, and FlexSnap plots overlap with the reference alignment. FlexSnap has 100 percent coverage of the reference alignment; there is a triangle in every circle.

FlexSnap alignments have 100 percent agreement on four structure pairs. One such pair is the alignment of NK-lysin (1nkl, 78 residues) with prophytepsin (1qdm, chain A, 77 residues). On this pair, all the sequential alignment methods (CE, DALI, FATCAT, and LGA) returned zero agreements. For the non-sequential ones: SARF returned 92%, MultiProt got 68%, and SCALI returned 69%. The reference alignment had 72 aligned pairs. As shown in Figure 5, the sequential alignment methods (only DALI and FATCAT shown) have their alignment paths along the diagonal and do not agree with the reference alignment (shown as circles).

### Sequential Flexible Alignments

Table 2 shows the alignments of different methods on the FlexProt dataset [11] which is obtained from the database of macromolecular motions[28]. We have implemented two versions of FlexSnap namely FlexSnap^*F*^, and ; In FlexSnap^*F*^, *C*(*P*_*j *_→ *P*_*i*_) is the cost of connecting *P*_*i *_with the rigid region to which *P*_*j *_belongs. In the second version, , *C*(*P*_*j *_→ *P*_*i*_) is the connection cost of *P*_*i *_with only *P*_*j*_. It is observed that when considering the entire rigid region, as in FlexSnap^*F*^, we get much better alignments, i.e., they have lower *rmsd *and fewer hinges. Moreover, FlexSnap^*F *^gives comparable results to the FATCAT method. In few cases, it got slightly shorter alignments with much better *rmsd *as in the case of the third and fourth alignment pairs.

**Table 2:** Comparison of FlexProt, FATCAT, FlexSnap^*F*^, and .

		FlexProt			FATCAT			**FlexSnap**^*F*^					
Pro1	Pro2	*l*	*r*	*T*	*l*	*r*	*T*	*l*	*r*	*T*	*l*	*r*	*T*
1wdnA(223)	1gggA(220)	218	0.94	2	220	1.01	2	220	0.96	2	220	0.96	2
1hpbP(238)	1gggA(220)	220	2.34	2	213	1.59	2	211	1.67	2	210	3.88	1
2bbmA(148)	1cll_(144)	139	2.22	1	144	2.28	1	138	1.8	1	138	1.80	1
2bbmA(148)	1top_(162)	147	2.40	3	145	2.28	3	137	1.78	3	137	1.78	3
1akeA(214)	2ak3A(226)	200	2.44	2	202	1.54	2	207	2.05	2	206	6.72	1
2ak3A(226)	1uke_(193)	182	2.90	2	188	2.97	0	184	2.36	1	184	3.08	0
1mcpL(220)	4fabL(219)	218	1.93	1	217	1.40	1	217	1.49	1	217	1.49	1
1mcpL(220)	1tcrB(237)	212	2.33	1	213	2.20	1	202	2.3	1	200	2.38	1
1lfh (691)	1lfg_(691)	691	1.41	2	686	0.89	2	688	0.99	2	688	0.99	2
1tfd (294)	1lfh_(691)	291	1.98	2	290	1.37	2	287	1.89	2	283	1.41	2
1b9wA(91)	1danL(142)	75	2.78	1	80	2.39	2	82	2.25	2	83	2.7	2
1qf6A(641)	1adjA(420)	323	4.43	1	351	2.68	1	326	2.45	3	320	2.47	2
2clrA(275)	3fruA(269)	253	2.71	2	245	3.06	0	254	2.57	3	252	4.31	0
1fmk (438)	1qcfA(450)	424	1.25	2	433	2.27	0	413	2.71	0	413	2.44	1
1fmk (438)	1tkiA(321)	231	3.28	2	238	3.07	0	241	2.58	3	242	3.14	2
1a21A(194)	1hwgC(191)	163	2.75	4	153	3.16	1	156	2.35	3	155	3.79	2

Comparison of FlexProt, FATCAT, FlexSnap^*F*^, and . Each alignment is reported in the following format: its length, *l*, its *rmsd*, *r*, and the number of hinges introduced, *T*.

### Flexibility in the DynDom Dataset

The DynDom dataset [25] is a comprehensive and non-redundant dataset of protein domain movements; it has been compiled by an exhaustive analysis of protein domain movements on all available protein structures using the DynDom program [29]. The protein conformations are first grouped into families based on sequence similarity, resulting in 1825 families with an average of 11.5 family members. Then a clustering procedure is applied to members of the same family to remove dynamic redundancy (same motion) and finally running the DynDom program to analyze domain movements in each family. There are currently 2035 representative pairs belonging to 1578 families in the DynDom dataset. Since these representative pairs involve domain movements, rigid alignment methods would not be able to align these pairs effectively, while flexible alignment methods will be able to introduce hinges and align the pairs more effectively. We define the coverage of the alignment as the percentage of the number of residues in the alignment to the length of the smaller protein. More formally, the coverage of an alignment of length *N*_*mat *_is defined as , where |*A*| is the length of protein *A*, similarly for |*B*|.

Table 3 shows the average coverage, *rmsd*, and hinges reported by different methods on the DynDom dataset. For the same structure pair, FlexProt reports different solutions with different number of hinges ranging from 0 to 5 hinges. For the sake of fair comparison, we choose the FlexProt alignment with the same number of hinges as the solution reported by FlexSnap. Moreover, we also run FlexSnap in rigid mode (FlexSnap^*R*^) with the number of allowed hinges set to 0 to investigate how it compares to rigid alignment methods. DALI has the highest coverage followed by FlexSnap. However, the average *rmsd *of FlexSnap alignments is much smaller than the average *rmsd *for DALI alignments. On average, FlexSnap introduced 0.59 hinges in the alignments. By introducing flexibility in the alignments, FlexSnap reported alignments with significantly smaller *rmsd *while maintaining high alignment coverage. Also when run in the rigid mode, FlexSnap^*R *^is competitive to state-of-the-art methods like DALI, Structal, and MultiProt.

**Table 3:** Comparison of several alignment methods on the DynDom dataset

DALI	Structal	MultiProt	**FlexSnap**^*R*^	FlexSnap	FlexProt
*aC*/*aR*	*aC*/*aR*	*aC*/*aR*	*aC*/*aR*	*aC*/*aR*/*aH*	*aC*/*aR*/*aH*

97/2.31	87/1.27	87/1.15	85/1.60	96/1.46/0.45	88/2.14/0.45

Two values are reported for the alignments of each method: average coverage for the method (*aC *in %), and average *rmsd *(*aR *in Å). For FlexSnap and FlexProt, we also report the average number of hinges introduced (*aH*). FlexSnap^*R *^is FlexSnap in rigid mode with the number of maximum allowed hinges set to zero.

#### DynDom Pairs with Low Coverage

**Figure 6:**
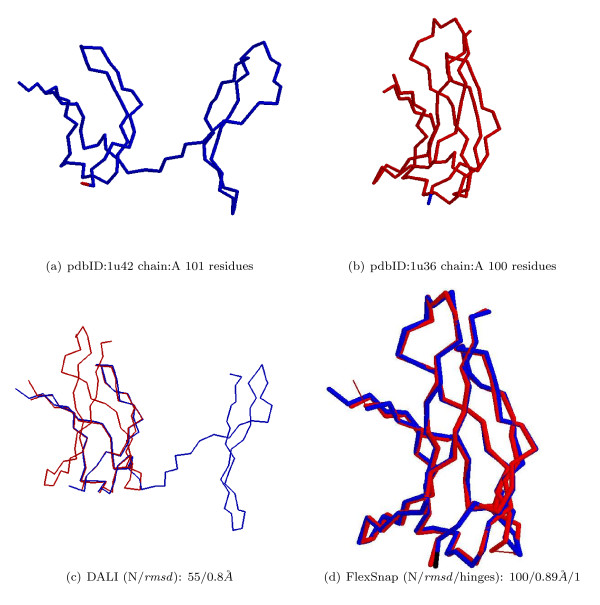
**An example of a rigid alignment with low coverage**. A DynDom pair with low alignment coverage: Rigid vs. Flexible alignment.

Rigid alignment methods try to optimize a score that is usually dependent on the length and *rmsd *of the alignment. Therefore, they might prefer shorter alignment with small *rmsd *over a longer alignment with significantly larger *rmsd*. In some cases, like when there is a movement in one of the proteins, they have no choice but to report a shorter alignment with an acceptable *rmsd *value. We analyze the alignments of structure pairs for which rigid alignment methods returned short alignments as compared to the length of the smaller protein. We run three different rigid alignment methods, DALI, Structal, and MultiProt, and get the pairs for which any of the methods returned a coverage less than or equal to 60%. The list has 30 pairs for DALI, 282 for Structal, and 164 for MultiProt. An example of a rigid alignment with low coverage is shown in Figure 6. For this DynDom pair, Structal reported an alignment of 52 residues with *rmsd *0.40 Å; MultiProt's alignment was 54 with *rmsd *0.52 Å.

Table 4 shows the average coverage, *rmsd*, and hinges reported by FlexSnap on these structure pairs. For fair comparison, we choose the FlexProt alignment with the same number of hinges as the FlexSnap solution. FlexSnap significantly improves the coverage of the alignments of these hard pairs. Moreover, it does so while maintaining good *rmsd *values and introducing on average about 1.5 hinges. In FlexSnap's scoring function, hinges are penalized and we only introduce a hinge if there is a significant increase in the alignment score. That explains why the number of hinges introduced is not large. DALI optimizes a score that incorporates the length and *rmsd *of the alignment. Thus for these 30 pairs, the score is too low for longer alignments, and thus DALI chooses to report shorter alignments with good *rmsd*, and thus low coverage on these 30 pairs. The Structal method reported low coverage alignments on many more structure pairs when compared to DALI. The reason behind that is the fact that the Structal method depends on the initial alignments for its initial transformations and it might miss the true alignment if the initial alignments are not good starting points.

**Table 4:** Comparison of FlexSnap and FlexProt on the DynDom pairs for which rigid alignment methods returned *coverage *≤ 60%

Rigid Alignment			FlexSnap	FlexProt
**Method**	**#Pairs**	***aC*(%)/*aR*(Å)**	***aC*(%)/*aR*(Å)/*aH***	***aC*(%)/*aR*(Å)/*aH***

DALI	30	31/2.3	89/1.75/1.37	79/2.36/1.37

Structal	282	52/0.77	94/1.72/1.34	93/2.08/1.34

MultiProt	164	53/1.12	92/1.59/1.56	93/2.0/1.56

Two values are reported for the alignments of each method: average coverage for the method (*aC *in %), and average *rmsd *(*aR *in Å). For FlexSnap and FlexProt, we also report the average number of hinges introduced (*aH*).

#### DynDom Pairs with Large rmsd

**Figure 7:**
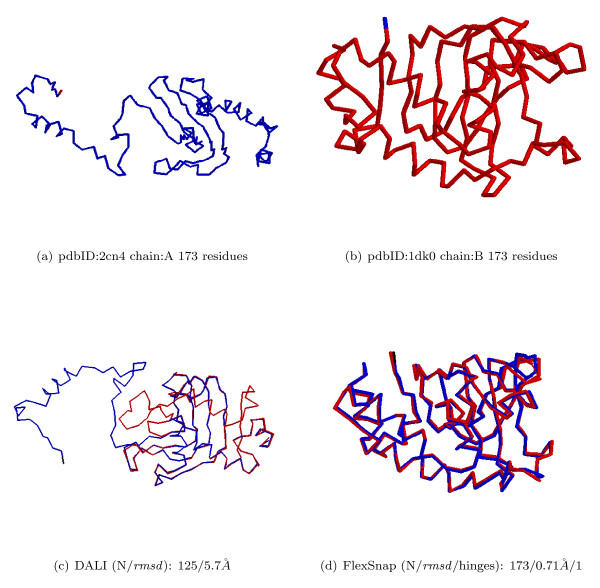
**An example of a rigid alignment with large *rmsd***. A DynDom pair with large alignment *rmsd*: Rigid vs. Flexible alignment.

In some cases rigid alignment methods would seek to optimize the score that favors longer alignments with acceptable *rmsd *values, and thus they may have good coverage on some pairs, but the *rmsd *values may be too large. Flexible alignments can be employed for these cases to get similar alignments but with much better *rmsd *values. For each of our test methods, namely DALI, Structal, and MultiProt, we compiled a list of the structure pairs for which the method reported an alignment with *rmsd *≥ 4.0 Å, and we ran FlexSnap on these pairs. An example of a rigid alignment with large *rmsd *is shown in Figure 7. FlexSnap reported an alignment with 100% coverage with an *rmsd *of 0.71 Å by introducing only one hinge in the alignment. Table 5 shows the average coverage, and *rmsd *as reported by the native rigid method and by FlexSnap. Under this criterion, DALI reported alignments with *rmsd *≥ 4.0 Å on 295 pairs, much more than what the other methods reported. MultiProt didn't report any alignment with large *rmsd*. In fact, all of the MultiProt alignments had *rmsd *≤ 2.3 Å; this can be explained by noting that MultiProt includes in the alignment only residue pairs which are closely aligned and thus the overall *rmsd *will not be large.

**Table 5:** Comparison of FlexSnap and FlexProt on the DynDom pairs for which rigid alignment methods returned alignments with *rmsd *≥ 4.0 Å

Rigid Alignment			FlexSnap	FlexProt
**Method**	**#Pairs**	***aC*(%)/*aR*(Å)**	***aC*(%)/*aR*(Å)/*aH***	***aC*(%)/*aR*(Å)/*aH***

DALI	295	94/5.89	94/1.61/1.54	93/2.02/1.54

Structal	16	88/5.03	82/1.97/2.19	78/2.75/2.19

Two values are reported for the alignments of each method: average coverage (*aC *in %), and average *rmsd *(*aR *in Å). For FlexSnap and FlexProt, we also report the average number of hinges introduced (*aH*).

FlexSnap significantly improved the average *rmsd *of the alignments of these pairs. For the 295 pairs for which DALI reported an average *rmsd *of 5.89 Å, FlexSnap reported an average *rmsd *of 1.61 Å. For the 16 pairs reported by Structal, FlexSnap average *rmsd *is 1.97 Å as opposed to 5.03 Å reported by Structal.

## Conclusions

We have introduced FlexSnap, a greedy chaining algorithm that reports both sequential and non-sequential alignments and allows twists (hinges). We assessed the quality of the FlexSnap alignments by measuring its agreements with manually curated non-sequential alignments (on the RIPC dataset). On the FlexProt dataset, FlexSnap was competitive to state-of-the-art flexbile alignment methods. Moreover, we demonstrated the benefits of introducing hinges by showing the significant improvement in the alignments reported by FlexSnap for the structure pairs for which rigid alignment methods reported alignments with either low coverage or large *rmsd *(on the DynDom dataset).

## Competing interests

The authors declare that they have no competing interests.

## Authors' contributions

SS and MJZ designed the FlexSnap algorithm with the proposed scoring scheme. SS coded the algorithm in C++ and wrote the paper. CB proposed the experiments and helped analyze the alignments. All authors read and approved the final manuscript.
